# Psychometric Properties of ADHD Rating Scale—5 for Children and Adolescents in Sudan—School Version

**DOI:** 10.3389/fpsyg.2022.883578

**Published:** 2022-06-27

**Authors:** Abdulkarim Alhossein, Abdulrahman Abdullah Abaoud, David Becker, Rashed Aldabas, Salaheldin Farah Bakhiet, Mohammed Al Jaffal, Manar Alsufyani, Nagda Mohamed Abdu Elrahim, Nouf Alzrayer

**Affiliations:** ^1^Department of Special Education, College of Education, King Saud University, Riyadh, Saudi Arabia; ^2^Department of Psychology, Chemnitz University of Technology, Chemnitz, Germany; ^3^Department of Psychology, College of Education, Sudan University of Science and Technology, Khartoum, Sudan

**Keywords:** ADHD Rating Scale—5, factor analysis, reliability, children and adolescents, psychometric properties

## Abstract

The ADHD Rating Scale—5 for Children and Adolescents, School Version, has been adopted and validated to be used in assessing ADHD among school children within Western contexts. However, there are few assessment tools in use for identifying ADHD characteristics in children in Sudan. Therefore, this study aimed to investigate the psychometric properties of this rating scale in the context of Sudan. To accomplish this, data were collected on a sample of 3,742 school-aged children and adolescents as reported by their teachers. Psychometric properties can be classified as very good, with very high reliability (>0.90), and high construct validity tested by exploratory and confirmatory factor analysis. Thus, the ADHD Rating Scale—5 for Children and Adolescents, School Version, is valid, reliable, and suitable to use for assessing ADHD symptoms among children and adolescents in the Sudanese context.

## Introduction

Attention-deficit hyperactivity disorder (ADHD) is a heterogeneous neuropsychiatric disorder that usually appears in childhood and is characterized by attention deficit, hyperactivity, and impulsivity (American Psychiatric Association, 2013). The disorder was identified in the early 20th century by doctors and educators who noticed that some children seemed bored and impulsive (Hahn and Morgan, 2015). It was initially included in the second edition of the *Diagnostic and Statistical Manual of Mental Disorders-II* (*DSM-II*; APA) in 1968, labeled as “Hyperkinetic Reaction of Childhood” (Adler et al., 2015). With the emergence of the fifth edition of the 2004 Guide (*DSM-5*), ADHD was henceforth fully included as a disorder of significant concern for children and adolescents (Guha, 2010).

ADHD has a generalized effect on an individual’s life and academic problems are common among students with ADHD (DuPaul et al., 2016a). The negative effects of ADHD can be distracting during the acquisition of academic knowledge and skills (Arnold et al., 2020) by affecting students’ attention to lessons, thus reducing their ability to learn and acquire information by traditional methods (Hahn and Morgan, 2015; Jeyanthi et al., 2018). Loe and Feldman (2007) indicated that pupils with ADHD have low reading and math scores, poor scores on tests in general, and a high probability of academic failure. Volpe et al. (2006) estimated the probability of academic performance problems as up to 80% among children with the diagnosis. Students with ADHD tend to obtain low scores on standardized reading, mathematics, and spelling tests (Morsink et al., 2021), and their achievement rate is about 25%–30% lower than the average (Sfrisi et al., 2017). Such problems appear early and continue through until late in the school experience (Bolic et al., 2013). In addition, the higher rate of academic problems in these students is associated with other challenges to academic success, such as failure, school dropout, and failures in secondary school tests (Reid, 2012). Overall, children with ADHD show a range of disturbing behaviors within the classroom (e.g., screaming, leaving one’s seat, whistling, etc.; Christiansen et al., 2015). These issues create additional difficulties, including low self-esteem, and poor academic performance (Primack et al., 2012), making these students more likely to fail at school (Mathunni et al., 2017). All of these factors make early and accurate diagnosis of ADHD extremely important, as the condition can have serious negative impact on academic performance (DuPaul et al., 2015).

The exact cause of ADHD is still unknown; both genetic and environmental factors are considered during diagnosis (Roigé-Castellví et al., 2021). In addition to neuropsychiatric and biological changes, social factors may affect the severity of the problems associated with the disorder (Christiansen et al., 2015). Persons with ADHD react swiftly and spontaneously to an above-average degree, with disrupted executive functions responsible for the proper emergence of behavior (Alloway, 2011). In addition, children with ADHD have difficulty determining whether a behavior is appropriate for the setting or thinking about the potential consequences of their actions, due to their impulsivity (Tan et al., 2018). This highlights the fact that ADHD is not the result of a lack of skills or knowledge, but rather a problem of engaging the appropriate reaction to a certain situation (Reid, 2012). Brain-imaging of the frontal lobe, the area of the brain associated with executive function, of children with ADHD has found that it is smaller in these individuals than average (Öztekin et al., 2021; Roigé-Castellví et al., 2021). In addition, working memory deficits that directly affect the learning process could also be a factor in why students with ADHD face greater difficulties in the academic environment related to their problem-solving, planning, and organization skills deficits (Daley and Birchwood, 2010; Rauch et al., 2012).

Specialists in the field of ADHD emphasize the importance of accurate diagnosis because of the advantages this creates for the individual with the disorder on a variety of levels (Barnes et al., 2020). For example, the Klasen (2000) study revealed that accurate diagnosis provided relief to parents of children with ADHD as it gave them a sense of control, reduced their feelings of guilt, and provided an incentive to be more active in seeking help for their child who might previously have been labeled as of low intelligence or been marginalized/punished for their disruptive behaviors (Bringewatt, 2011). To obtain an accurate diagnosis of ADHD, the diagnostician looks for the frequent occurrence of certain symptoms, including attention deficits and/or hyperactivity and impulsiveness, to a degree that interferes with the individual’s social, academic, or professional performance (Wernersson et al., 2020).

According to the *DSM-5*, diagnosing ADHD in children depends on a set of strict criteria; specifically, at least six of the established symptoms of inattentiveness or at least six of the established symptoms of hyperactivity and impulsiveness must be recognized and persist over the course of 6 months or more (Mathunni et al., 2017; Wernersson et al., 2020). Additionally, the symptoms must appear in two or three situations and before the age of 12 (National Health Service, 2021). These criteria are required so as to differentiate ADHD from other disorders with which it shares certain symptoms (e.g., developmental disability, learning disorders, autism, depression, anxiety disorder; Adler et al., 2015).

Systematic reviews of the literature have found that little research has been conducted on ADHD in Arab societies (e.g., Farah et al., 2009). Furthermore, while more recently researchers have noted an increase in such research in Arab countries, it is still less than desirable or competitive with investigations in western context, and the methods and procedures used limit the generalizability of the findings of the research that has occurred (Alkhateeb and Alhadidi, 2016). In Sudan, there is a scarcity of up-to-date diagnostic tools that have been adapted to the local environment. This is a matter of concern since the estimated prevalence of ADHD in Sudan is around 9.4% (Osman et al., 2015) to 12.6% (Haroun, 2010), while the estimated prevalence in Africa as a whole is 7.47% (Ayano et al., 2020). This higher incidence rate further emphasizes the importance of offering appropriate and validated diagnostic tools in Sudan.

Al-Awad and Sonuga-Barke (2002) conducted the first study in Sudan to assess the psychometric properties of a version of the Conners Rating Scales, using the CTRS-39 (Conners’ Teaching Rating Scale) and the CPRS-48 (Conners’ Parent Rating Scale), which they had first translated into Sudanese Arabic; the translation was confirmed utilizing a back-translation check. Teachers and parents applied the scales on a normative sample that included 300 families with children aged from 6 to 10 years of age. The two versions of the scale demonstrated high levels of reliability and internal consistency. Several years later, Madani (2007) conducted a psychometric study on the Attention Deficit/Hyperactivity Disorder Test (ADHDT; Conners, 1990), to identify ADHD in those between the ages of 6 and 14 in the state of Khartoum. First, the scale was translated and then items were modified, deleted, and merged. Next, the scale was implemented on a sample of 404 students. The results indicated high reliability with a Cronbach’s alpha = 0.84.

Some Sudanese researchers developed ADHD-questionnaires based on the *DSM-III* (American Psychiatric Association, 1980) or *DSM-IV* (American Psychiatric Association, 1994; e.g., Fathy, 2010; Unpublished master’s thesis^1^; Khalaf-Allah, 2011, 2013, 2016; Al Hassan, 2017; Unpublished master’s thesis^2^; Ahmed and Khalaf-Allah, 2018; Taha, 2020). However, the samples in these studies were quite small. Other studies used questionnaires developed in Egypt based on the *DSM-IV* (American Psychiatric Association, 1994). For example, the questionnaire developed by Al-Nubi (2010) was used in two studies (Al-Hussein, 2015; Unpublished master’s thesis^3^; Abdel-Qader, 2018; Unpublished master’s thesis^4^); and, a questionnaire developed by Al-Desouki (2007) was used in the work of Awad Allah and Ajbna (2013). These three studies used Egyptian-developed questionnaires in Sudan, after verifying their validity and reliability, but again they involved only small samples and did not include all the age groups of childhood and adolescence.

Different types of studies were conducted by medical researchers, such as Mohammed (2014); Osman et al. (2015); and Ahmed and Mohamed (2015). However, these studies did not aim to standardize or identify psychometric properties for rating scales but rather were conducted to identify the prevalence of ADHD or identify the roles of parents. They were still notable as they employed international rating scales for the first time in Sudanese studies, although again the sample sizes were small or the researchers did not provide sufficient information about the samples. Mohammed (2014) used the Weiss Functional Impairment Rating Scale – Parent (WFIRS–P) with a sample of 120 individuals who visited clinics. The scale was translated into Arabic by an English-Arabic translator, checked *via* back-translation, and validated for Sudanese culture. Osman et al. (2015) applied the SNAP-IV-C rating scale filled by teachers and parents. A preliminary study was conducted in which 50 students from a separate school participated to validate this scale. Unfortunately, the study did not provide detailed information on how the validity and reliability results were obtained. Ahmed and Mohamed (2015) used the Attention Deficit/Hyperactivity Test, developed by Gilliam in 2010, but failed to provide information about the psychometric properties or the preliminary sample, except that participants were between the ages of 5–17 years old. Al-Awad (2019) conducted a study to assess the conceptual and linguistic equivalence of the translations of the revised Conners’ Teacher Rating Scale-28 (CTRS-28) and the modified Conners Parent Rating Scale-48 (CPRS) to identify 200 children. The results indicated that the translations of the two revised scales were reliable and valid tools for measuring behavioral and emotional disorders in children in urban areas of central Sudan.

The literature review on measures of ADHD in the Sudanese context revealed many gaps, including the use of outdated tools, the use of local tools that do not have the advantages of international standards, the small numbers of the samples and their restriction to selective age groups (children/adolescents only) that were used to assess the psychometric properties, limited procedures used to assess the psychometric properties, and the lack of psychometric information. This study aimed to address these gaps by utilizing the ADHD Rating Scale—5 (DuPaul et al., 2016b), an actual scale for ADHD based on the criteria of the *DSM-5*, with a large and representative sample of children and adolescents to assess its psychometric properties.

Existing theories about the factor structure behind the 18 symptom items of the ADHD Rating Scale—5 are based on the assumption of a non-hierarchical structure with two to three correlated factors for inattention, hyperactivity, and impulsivity (Baumgaertel et al., 1995; Pillow et al., 1998; Molina et al., 2001; Arildskov et al., 2020). Additionally, Power et al. (2017) extracted six highly correlated factors (academic dim.; behavioral dim.; self-esteem dim.; inattention; hyperactivity-impulsivity, teacher relations dim.) for the 12 impairment items of the ADHD Rating Scale—5, where on each two of the items are loaded to each of the six factors. In the following, the researchers attempt to replicate these structures in the Sudan sample using confirmatory factor analysis, while also investigating alternative structures to identify any cultural or ethnic specifics.

## Materials and Methods

### ADHD Rating Scale—5

The ADHD Rating Scale—5 (DuPaul et al., 2016b) is an 18-item scale for the assessment of children that incorporates the symptoms of ADHD established in the *DSM-5* (American Psychiatric Association, 2013). The *symptom scale* includes two subscales, one for *inattention* (short: “Inatt.”) and one for *hyperactivity-impulsivity* (short: “Hyp.-Imp.”). The *Inattention* items are: (1) Attention to detail; (2) Sustaining attention; (3) Does not seem to listen; (4) Follows instructions; (5) Difficulty organizing; (6) Sustained mental effort; (7) Loses things; (8) Distracted; (9) Forgetful. The *Hyperactivity-impulsivity* items are: (1) Fidgets; (2) Leaves seat; (3) Runs about; (4) Playing quietly; (5) On the go; (6) Talks excessively; (7) Blurts out answers; (8) Awaiting turns; (9) Interrupts or intrudes. Each item is responded to using a four-point Likert scale, where 0 = “never or rarely”; 1 = “sometimes”; 2 = “often”; and 3 = “very often.” Total scores for (sub)scales are to be formed using summations of all associated items.

The ADHD Rating Scale—5 also includes six items for *impairment*, again on the two subscales of *Inattention* and *Hyperactivity-Impulsivity*. These items are (1) Family/Teacher Relations; (2) Peer Relations; (3) Homework Functioning; (4) Academic Functioning; (5) Behavioral Functioning; (6) Self-Esteem. These six items were rated twice, after rating *Inattention* and after rating *Hyperactivity-Impulsivity*. Ratings and scorings followed the same procedure as for the *symptom scale*, however, on this four-point Likert scale, 0 = “no problem”; 1 = “minor problem”; 2 = “moderate problem”; and 3 = “severe problem.”

Using this scale, teachers can assess a student’s behavior for ADHD in school and parents can assess the child’s behavior for ADHD at home. Differences in mean ratings between these two types of assessors was found to especially occur for *Hyperactivity-Impulsivity*; however, only marginal differences occurred in factor structures, with somewhat higher loadings in ratings by teachers compared to ratings by parents (DuPaul et al., 2016b). For this study, parents and teachers created the ratings and analyses of both datasets were done. However, due to space limitations here, the analysis results are presented in the text for the teachers’ dataset only, while the results from the parents’ dataset are included in the Appendix for comparison purposes only.

### Participants

To assess the psychometric properties of the ADHD Rating Scale—5 for Children and Adolescents—school version (DuPaul et al., 2016b) in Sudan, descriptive research design was used in this study (Cohen et al., 2007). The total sample consisted of 3,742 kindergarten- and school-aged children from 5 to 17 years old attending schools in the metropolitan area of Khartoum. The sample was selected using stratified random sampling. The selected schools are representative of the area and include students of different social, economic, cultural, age ranges, and grade levels across the seven regions of the country. Of the total group, 288 (34.4%) were from Khartoum City, 1,012 (27.0%) were from the city of Bahri, and 1,442 (38.5%) were from the city of Omdurman. The gender-ratio was well-balanced with 1,947 (52.0%) males and 1,795 (48.0%) females. Of the 3,742 participants, there were 56 (1.5%) children who had already been diagnosed with ADHD. Ninety-two (2.5%) of the participants had been diagnosed with another type of disability, as follows: (a) autism—37% or 0.99% of the total pool; (b) intellectual disability—35% or 0.94% of the total pool; (c) learning disability—34% or 0.91% of the total pool; (d) Deaf/Hard of Hearing (D/HH)—20% or 0.53% of the total pool; and (e) significant visual impairment—20% or 0.53% of the total pool. Additionally, 620 (16.6%) of the participants had been identified as gifted.

The children were assessed by their teachers, who were 3,742 professionals asked to rate Student Number 5 on the list of students in their individual class. The teachers ranged in age from 22 to 73 years old (*M* = 42.46; *SD* = 10.91); 833 (22.3%) obtained a high school diploma or less, 2,196 (58.7%) had a bachelor’s degree, 675 (18.0%) had a master’s degree, and 38 (1.0%) did not specify their educational background. Of these teachers, 2,887 (77.2%) taught general education and 736 (19.7%) taught special education, while others (119, 3.2%) did not specify an area of specialization. In terms of length of teaching experience, 620 (16.6%) had less than 5 years, 871 (23.3%) had between 5 and 10 years, 2,227 (59.5%) had more than 10 years, and 25 (0.6%) did not specify how much experience they had. At least one special program (professional development) has been attended by 1,010 (27.3%) of the teachers, 340 (9.1%) attended two, 91 (2.4%) attended three, and 141 (3.8) indicated attending more than three such programs.

### Data Collection Procedures

Since Sudan is an Arab country whose culture and language are somewhat similar to that of Saudi Arabia, the Arabic version of the ADHD Rating Scale—5 for Children and Adolescents—School Version (DuPaul et al., 2016b), was administered to collect data for assessing the reliability and validity of the scale in Sudan. This scale was translated into Arabic and validated in Saudi Arabia by Alhossein and Bakhiet (2017).

After obtaining the approval from the Ministry of Education in Sudan and the University of Khartoum, a group of 35 graduate students in the College of Education attended a workshop to introduce the scale and explain how to complete, correct, and grade it; they were also trained on how to introduce teachers to and complete the scale. The 35 graduate students were divided into seven groups of five to contact schools located in the seven regions for recruiting potential participants. The potential participants were provided with information about the purposes of the study, how to participate, and the Informed Consent form along with a copy of the scale to complete upon agreement to participate. The participants were informed that participation was voluntary and they were free to withdraw from the study at any time. The data collection took a period of 2 months. At the end, data had been obtained for a total of 3,742 cases, 3,736 (>99%) of which had full data. There were never more than two cases of missing values per variable and only symptom items were affected.

### Coding

Scores from the ADHD Rating Scale—5 were not recoded, thus higher scores represent a higher frequency of symptoms or problems, with “0” as the minimum and “3” (items), 27|18 (symptoms|impairment subscales) and 54|36 (symptoms|impairment total scores) as the maximum. Within the recorded data, 20 datapoints gave an invalid score of “4.” It is not clear whether these were coding errors or due to incorrectly completed questionnaires. However, as these 20 datapoints came from only four different raters—who also gave some valid answers—the latter was assumed. Thus, the researchers decided to replace all scores of “4” with a “3.” Overall, however, due to the sample size, this did not cause significant deviations.

### Statistics and Analyses

Reliability analyses, factor analyses [exploratory factor analysis (EFA) and confirmatory factor analysis (CFA)], and multiple regression analyses (MRA) were applied on the results from both the symptom and the impairment scale. In case of EFA, the researchers used maximum likelihood for factor extraction assuming multivariate normal distribution, and obliging for rotation allowing intercorrelations. CFA was run with maximum likelihood estimation (ML). Due to the extremely low percentage of missing values, the researchers dispensed with imputation procedures and used the complete case approach for the factor analyses. All analyses were done with R (version 4.0.0; R Core Team, 2020). The package Psych (version 2.1.6; Revelle, 2019) was used for the reliability analyses (Cronbach’s *α*; Guttman’s 6; McDonald’s *ω*); for the factor analyses, the packages Lavaan (version 0.6–7; Rossel, 2012), psy (version 4.0.3; Falissard, 2012), ltm (version 1.1–1; Rizopoulos, 2018) and nFactors (version 2.4.1; Raiche and Magis, 2020). Settings and results from all analyses done with R can be viewed using the data and the files RSYNTEA.txt and ROUTTEA.txt in the Supplementary Material. For the results based on the ratings by parents, please see RSYNPAR.txt and ROUTPAR.txt in the Supplementary Material.

## Results

### Reliability

Cronbach’s *α* were calculated for a general factor, but, as two underlying factors are assumed for the symptom items in the manual (DuPaul et al., 2016b), the scales Inatt. and Hyp.-Imp. were analyzed separately (see Tables 1, 2). As the real factor structure is so far unknown, McDonald’s *ω* was added as an alternative analysis. However, reliability is consistently high for symptom items (*α*_Inatt._ = 0.92; *ω*_Inatt._ = 0.93; *α*_Hyp.-Imp._ = 0.90; *ω*_Hyp.-Imp_ = 0.91) and impairment items (*α*_Inatt._ = 0.87; *ω*_Inatt._ = 0.91; *α*_Hyp.-Imp._ = 0.87; *ω*_Hyp.-Imp_ = 0.92). Confidence intervals (95%) are within a maximum of one decimal place and dropping items does not result in any significant changes.

**Table 1:** Reliability for the symptom items and item drop of the ADHD Rating Scale—5.

Item	*N*	Cronbach’s *α*						McDonald’s *ω*	
		Raw	Std	95% CI		G6	*M_r_*	Hierarch.	Total
				Lower	Up.				
Drop: attention to details	3,742	0.91	0.91	–	–	0.90	0.56	–	–
Drop: sustaining attention	3,742	0.91	0.91	–	–	0.90	0.55	–	–
Drop: does not seem to listen	3,742	0.91	0.91	–	–	0.90	0.56	–	–
Drop: follow instructions	3,741	0.91	0.91	–	–	0.90	0.55	–	–
Drop: difficulty organizing	3,742	0.91	0.91	–	–	0.90	0.55	–	–
Drop: sustained mental effort	3,740	0.91	0.91	–	–	0.90	0.56	–	–
Drop: loses things	3,742	0.91	0.91	–	–	0.90	0.57	–	–
Drop: distracted	3,742	0.91	0.91	–	–	0.90	0.56	–	–
Drop: forgetful	3,742	0.91	0.91	–	–	0.90	0.56	–	–
Inatt. total	3,742	0.92	0.92	0.91	0.92	0.91	0.56	0.85	0.93
Drop: fidgets	3,742	0.89	0.89	–	–	0.89	0.52	–	–
Drop: leaves seat	3,742	0.89	0.88	–	–	0.88	0.49	–	–
Drop: runs about	3,742	0.89	0.88	–	–	0.88	0.50	–	–
Drop: playing quietly	3,742	0.90	0.89	–	–	0.89	0.52	–	–
Drop: on the go	3,742	0.89	0.88	–	–	0.88	0.49	–	–
Drop: talks excessively	3,741	0.89	0.88	–	–	0.88	0.50	–	–
Drop: blurts out answers	3,740	0.89	0.88	–	–	0.88	0.51	–	–
Drop: awaiting turns	3,742	0.89	0.88	–	–	0.88	0.50	–	–
Drop: interrupts or intrudes	3,742	0.89	0.88	–	–	0.88	0.51	–	–
Hyp.-Imp. Total	3,742	0.90	0.90	0.90	0.91	0.90	0.50	0.81	0.91

Analyses were done for Inatt. and Hyp.-Imp. separately; A = Inatt., H = Hyp.-Imp.; *N* varies due to missing/false values; reliability for Inatt. and Hyp.-Imp.; lower and upper *α* for 95% confidence boundaries; G6 = Guttman’s 6; M_r_ = mean intercorrelation; results for items: if dropped.

**Table 2:** Reliability for the impairment items and item drop of the ADHD Rating Scale—5.

Item	*N*	Cronbach’s *α*						McDonald’s *ω*	
		Raw	Std	Lower	Upper	G6	*M_r_*	Hierarch.	Total
Drop: teacher relations	3,742	0.85	0.85	–	–	0.83	0.51	–	–
Drop: peer relations	3,742	0.84	0.84	–	–	0.83	0.50	–	–
Drop: academic functioning	3,742	0.84	0.84	–	–	0.81	0.50	–	–
Drop: behavioral funct.	3,742	0.84	0.84	–	–	0.82	0.50	–	–
Drop: homework funct.	3,741	0.84	0.84	–	–	0.83	0.50	–	–
Drop: self-esteem	3,742	0.85	0.85	–	–	0.84	0.50	–	–
After Inatt. rating	3,742	0.87	0.87	0.86	0.87	0.86	0.50	0.78	0.91
Drop: teacher relations	3,742	0.86	0.86	–	–	0.85	0.52	–	–
Drop: peer relations	3,742	0.85	0.85	–	–	0.84	0.50	–	–
Drop: academic functioning	3,741	0.84	0.85	–	–	0.83	0.52	–	–
Drop: behavioral funct.	3,742	0.84	0.85	–	–	0.83	0.51	–	–
Drop: homework funct.	3,742	0.85	0.85	–	–	0.84	0.51	–	–
After Hyp.-Imp. rating	3,742	0.87	0.87	0.86	0.88	0.87	0.51	0.76	0.92

Analyses done for Inatt. and Hyp.-Imp. separately; A = Inatt., H = Hyp.-Imp.; *N* varies due to missing/false values; reliability for Inatt. and Hyp.-Imp.; lower and upper *α* for 95% confidence boundaries; G6 = Guttman’s 6; M_r_ = mean intercorrelation; results for items: if dropped.

### Factor Analyses for Symptom Items

To analyze the underlying factor structure of the symptom items for Sudanese children, the researchers first ran explorative factor analysis. Assuming correlation of the extracted factors and missing normal distribution in the items, the researchers used an oblique rotation (oblimnin) and maximum likelihood (ml) for extraction. The prerequisites for this are met by the Sudanese data, with exceptional Kaiser–Meyer–Olkin (KMO) test results of 0.96 and significant Barlett-sphericities (*χ*^2^[153] = 37366.31; *p* < 0.001) for symptom items. The researchers found that EFA suggested a one-factor solution based on the accelerated factors approach, and a two-factor solution based on the optimal coordinates approach and Velicer’s minimum average partial (MAP) test. However, fit (RMSR = 0.04) were good for the latter but not for the former (RMSR = 0.09). Also, the two-factors solution explains an additional 10% of the total variance compared to the one-factor solution (*R*^2^ = 0.54 vs. 0.44). The three-factors solution increases the *R*^2^ to 0.61, but shows the same fits (RMSR = 0.04) as the two-factors solution. Thus, Table 3 shows factor loadings for the two-factors solution only. The pattern of loadings distinguishes clearly between the two dimensions Inatt. and Hyp.-Imp., with no cross-loadings.

**Table 3:** EFA-results for the symptom items of the ADHD Rating Scale—5.

Items	Cmnl.	Factor loadings (λ)	
		C1	C2
Attention to details	0.54	0.77	−0.07
Sustaining attention	0.63	0.78	0.02
Does not seem to listen	0.54	0.66	0.11
Follow instructions	0.62	0.81	−0.04
Difficulty organizing	0.61	0.77	0.01
Sustained mental effort	0.55	0.79	−0.06
Loses things	0.49	0.66	0.07
Distracted	0.54	0.62	0.16
Forgetful	0.55	0.73	0.02
Fidgets	0.42	0.09	0.58
Leaves seat	0.57	0.08	0.70
Runs about	0.55	0.01	0.74
Playing quietly	0.42	0.18	0.52
On the go	0.59	0.03	0.75
Talks excessively	0.55	−0.04	0.77
Blurts out answers	0.50	−0.10	0.77
Awaiting turns	0.51	0.00	0.72
Interrupts or intrudes	0.48	0.02	0.68
*R* ^2^		0.53	0.47

Cmnl. = communalities; rotation = oblimin; extraction = Maximum Likelihood; *N* = 3,736; RMSR = 0.03.

**Figure 1:**
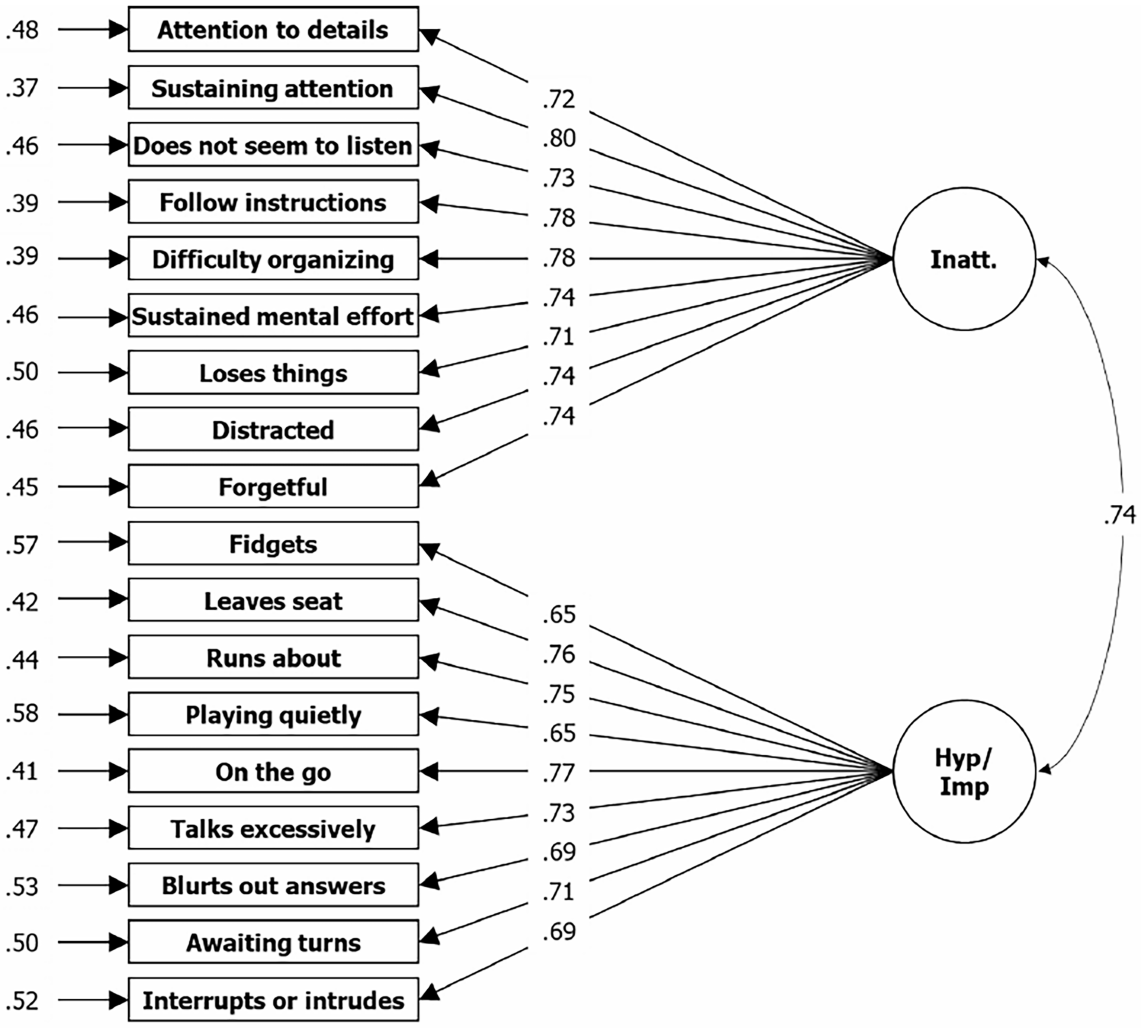
Results from CFA for two factor solution based on symptom items of the ADHD Rating Scale—5. *N* = 3,736; Loglikelihood user model (H_0_) = −58729.958; Fits: *χ*^2^[135] = 2135.909, CFI = 0.946, TLI = 0.939, RMSEA = 0.063, SRMR = 0.037; left = residual variances, center = factor loadings (lambda), right = correlation.

**Figure 2:**
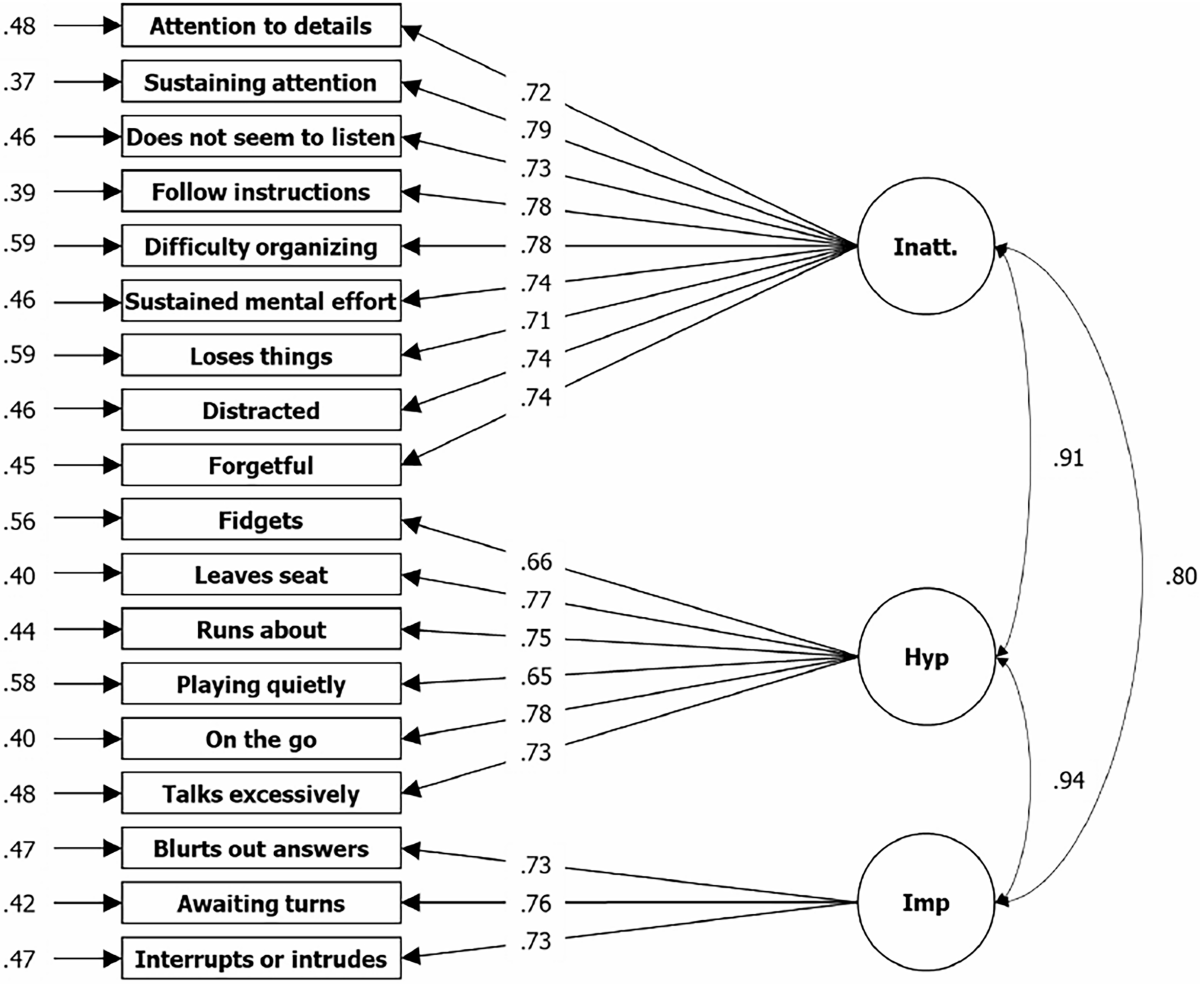
Results from CFA for three factor solution based on symptom items of the ADHD Rating Scale—5. *N* = 3,736; Loglikelihood user model (H_0_) = −58601.647; Fits: *χ*^2^[135] = 1879.287, CFI = 0.953, TLI = 0.946, RMSEA = 0.060, SRMR = 0.035; left = residual variances, center = factor loadings (lambda), right = correlation.

The researchers ran a CFA assuming two correlating factors without an additional first order factor (see Figure 1). The decision to accept or reject the model was less clear this time. The log-likelihood of the specified model (H_0_) argues for a rejection, with values of −58749.227. Goodness of fit indices are partly sufficient and partly insufficient, with *χ*^2^[134] = 2131.909 (*p* < 0.001), CFI = 0.946, TLI = 0.939, RMSEA = 0.063, and SRMR = 0.037, but marginal and to be assessed as sufficient for the overall picture. Compared to the manual (there: Figure 2), *λ* are overall lower in our results, specifically between 0.71 and 0.80 for Inatt. compared to 0.90 to 0.94 in the manual, and between 0.65 and 77 for Hyp.-Imp. compared to 0.87 to 0.94 in the manual. Finally, the log-likelihood of a specified three-factor model (H_0_) shows a value of −58691.647, but *χ*^2^[135] = 1879.287 (*p* < 0.001), CFI = 0.953, TLI = 0.946, RMSEA = 0.060, and SRMR = 0.035, and therefore an overall sufficient fit (see Figure 2). For a more detailed comparison of the CFA results of the three models, please reference Table A5 in the Appendix. Fit indices are similar if models have been run with males and females separately, even if they were consistently slightly better for females (see Table A6 of the Appendix).

### Factor Analyses for Impairment Items

KMO test results for impairment items resulted in a very good overall-MSA of 0.91 and significant Barlett-sphericities (*χ*^2^[66] = 27115.12; *p* < 0.001) for impairment items. Here, EFA suggested a one-factor solution based on the accelerated factors approach, and a two-factor solution based on the optimal coordinates approach and Velicer’s MAP. RMSR was 0.08 for the one-factor solution and 0.05 for the two-factors solution. However, RMSR was significantly better at 0.01 for six sufficient factors, as recommended by the manual (there: Figure 2). The cumulative explained variance is 0.57 for the two-factor solution, 0.50 for the single-factor solution, and 0.73 for the six-factors solution. Thus, the latter was determined to be the most appropriate; factor loadings for the six-factors solution are shown in Table 4. In some places, two items load on one of the six factors (IA1 and IH1 on C1, IA2 and IH2 on C3, IA3 and IH3 on C4, IA4 and IH4 in C2), as the theory assumes. However, item pairs IA5/IH5 and IA6/IH6 both load on C5, whereas C6 only explains two additional percentages of the overall variance.

**Table 4:** EFA-results for the impairment items 25 of the ADHD Rating Scale—5.

Items	Cmnl.	Factor loadings (λ)					
		C1	C2	C3	C4	C5	C6
Attention to details	0.56	0.39	0.03	0.31	0.16	−0.02	0.16
Sustaining attention	0.99	−0.02	0.02	1.00	0.00	0.00	0.00
Does not seem to listen	0.76	0.01	−0.04	0.03	0.89	0.00	−0.02
Follow instructions	0.69	0.02	0.14	−0.03	0.66	0.07	0.13
Difficulty organizing	0.64	0.03	0.00	0.10	0.13	0.55	0.31
Sustained mental effort	0.54	0.01	−0.02	0.08	0.14	0.57	0.16
Fidgets	1.00	1.01	0.01	−0.02	−0.01	0.00	0.00
Leaves seat	0.63	0.29	−0.06	0.29	0.06	0.33	−0.22
Runs about	0.72	0.07	0.29	0.03	0.49	0.07	−0.23
Playing quietly	1.00	0.01	0.96	0.02	0.02	0.02	0.01
On the go	0.62	0.07	0.14	0.02	−0.05	0.68	0.00
Talks excessively	0.62	0.01	0.04	−0.01	0.03	0.74	−0.15
*R* ^2^		0.17	0.15	0.17	0.22	0.25	0.03

Cmnl. = communalities; rotation = oblimin; extraction = Maximum Likelihood; *N* = 3,742, R^2^_cumul._ = 0.73, RMSR = 0.01.

**Figure 3:**
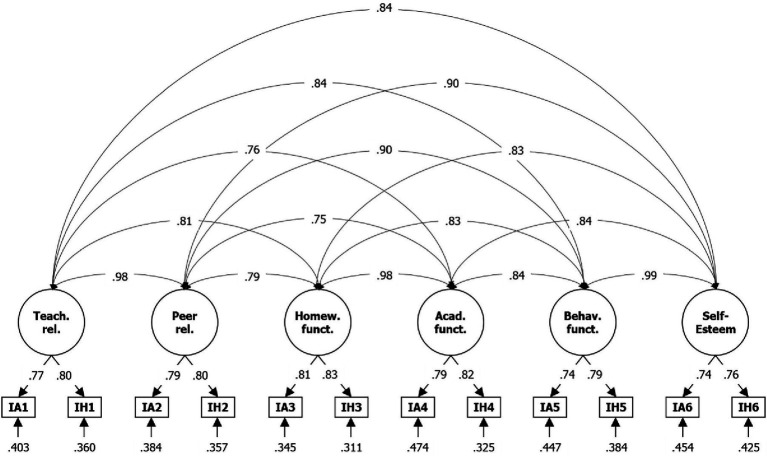
Results from CFA for six-factor solution based on impairment items of the ADHD Rating Scale—5. IA = impairment item filled after Inatt. assessment, IH = impairment item filled after Hyp.-Imp. assessment; *N* = 3,742; Loglikelihood user model (H_0_) = −35353.921; Fits: *χ*^2^[39] = 1876.112, CFI = 0.932, TLI = 0.885, RMSEA = 0.112, SRMR = 0.035; bottom = residual variances, center = factor loadings (lambda), top = correlation.

In CFA, the log-likelihood of the specified six-factors model (H0) argues for a rejection, with a value of −35353.921 (see Figure 3). Goodness of fit indices are partly sufficient and partly insufficient, with *χ*^2^[134] = 1876.112 (*p* < 0.001), CFI = 0.932, TLI = 0.885, RMSEA = 0.112, and SRMR = 0.035, which thus, in the Sudanese case, results in their being assessed as insufficient in the overall picture. Again, *λ* are lower compared to the manual, at between 0.74 and 0.83 compared to 0.93 and 0.99. Moreover, fit indices were again consistently slightly better—but similar—for females than males (see Table A6 of the Appendix).

## Discussion

Few scales are in use in Sudan for the assessment of ADHD in school-aged children and adolescents. There are also no standardized measures for assessing ADHD that are based on the *DSM-5* for use in public schools. The current study aimed to examine the validity and reliability of the ADHD Rating Scale—5 for Children and Adolescents, School Version in the Sudanese context. Compared to other studies that have investigating assessing ADHD in this population in Sudan, one strength of this study is that the data were collected from a large number of children and adolescents with and without ADHD at all grade levels of public education in Sudan. This provides greater value to the results and translates to a better contribution to the understanding and evaluation of developmental symptoms in Sudanese children and adolescents with ADHD. Another strength is the demographics of the participants with and without ADHD, which represented all components of Sudanese society. Furthermore, this research involved the application of the school version of the scale as completed by Sudanese teachers. When evaluating a student, teachers could observe behaviors in the classroom as compared to those of their peers, which is not possible when evaluation is implemented by parents who do not have access to numbers of typical students with whom to compare their child. This highlights the importance of the use of teacher rating scales (Kovshoff et al., 2012; DuPaul et al., 2014; Berri and Al-Hroub, 2016).

In this study, several important points were made regarding the ADHD Rating Scale—5, which consistently showed good validity and reliability indices during this research. This finding concurs with those of the studies conducted by Alhossein and Bakhiet (2017); Dobrean et al. (2021), and DuPaul et al. (2016b), all of which reported the ADHD Rating Scale—5 as having good psychometric properties samples of children and adolescents. Our results showed that an Arabic version of the ADHD Rating Scale—5 also has good psychometric properties, maintaining the quality of the original version. Reliability is very good in our Arab version, as is consistent with the results of the studies conducted by Alhossein and Bakhiet (2017); Dobrean et al. (2021), and DuPaul et al. (2016b). The construct validity of the symptom scale was confirmable by factor analyses, which showed the factor structure is similar to that found in western samples, even if the factor loadings are slightly lower and residual variances are higher. These characteristics might be due to the teachers in the present study having less familiarity with psychometric measurement methods as well as larger class sizes, which might mean that it is more difficult for individual teachers in the study to focus on one child for the purposes of completing the scale. Also, as shown by Husain et al. (2019) which involved a sample of children from the same region, school attendance in Sudan is more selective and the age distribution within grades or classes shows higher diversity. In Sudan, only ~22% of boys and 23% of girls aged 36–59 months attend pre-school education. Similarly, only 36% of boys and 38% of girls start primary education at the standard school entry age of 6 years old (Central Bureau of Statistics and UNICEF Sudan, 2016). Overall school enrollment in the country was 47% in 2017 (UNESCO Institute for Statistics, 2017). While these numbers might be somewhat higher in the urbanized area that was the setting of this study, nevertheless, enrolment figures in Sudan tend to fall below those of western countries. This issue leads to a sample that is less representative of the general population.

The researchers found the largest deviation to results from other studies in relation to the impairment scale. According to EFA, the first four factors (Teacher relations; Peer relations; Homework functioning; Academic functioning) could be clearly identified, but items for Behavioral functioning and Self-Esteem loaded on a common rather than two separate factors. However, both domains are defined as related to disruptive behavior, the first directly and the latter indirectly by a various level of discipline due to disruptive behavior. Therefore, it seems appropriate to distinguish between the two factors in terms of content and to load the associated items together on a common factor.

In summary, the current version of ADHD Rating Scale-5 was found to have appropriate psychometric characteristics in the Sudanese educational environment, which was supported by the types of validity and reliability established in this study. The current study provided more cross-cultural evidence for the effectiveness of the ADHD Rating Scale-5. It therefore can be used to assess ADHD symptoms among Sudanese children and adolescents. It is a useful tool to assess students for identifying ADHD symptoms. The availability of such an important tool can enable teachers and caregivers to act as specialists in conducting regular assessment of children and adolescents prior to the final assessment. It can also enable school professionals to conduct initial assessments to improve their educational interventions for children and adolescents with ADHD in Sudan. Finally, it can be of great benefit in keeping track of the changes that appear on children and adolescents with ADHD and monitoring the effects of the interventions provided to them.

### Limitations and Implications

This study involved a relatively large sample (3,742) of Sudanese children and adolescents aged from 5 to 17 years old in all school grade levels in public schools affiliated with the Ministry of Education in the country’s capital of Khartoum. The study was limited in that it was specifically focused on only establishing the validity and reliability of the ADHD Rating Scale—5 for Children and Adolescents, School Version in Sudan, rather than in a more generalized context with a more diverse sample. Despite the study’s large sample, it was limited in that only the school version of the scale was used and validated. The home version of the scale needs to be explored in future studies. It would also be useful to conduct a future study to obtain normative data for the ADHD Rating Scale—5, as well as its differential validity, since the researchers in this study only established confirmatory and exploratory factor validity.

A notable limitation of this research is the matter of the partly unsatisfactory fit indices, specifically in the single-factor model for the symptom items. This issue was striking in that it seems to have been completely caused by the male subsample, especially as this sample is somewhat larger, thus should be suspected to gives the better fit. One possible explanation is that the teachers did not rate girls and boys with equal accuracy, possibly due to cultural factors. Overall, the researchers did not make any *ad hoc* adjustments to the models for fit-improvement, as such efforts would only have affected one of the three models and in the remaining two models, the fit indices are close to their critical value.

Several implications can be drawn from the findings of this study. First, it is important to make the ADHD Rating Scale—5 available in public schools in Sudan to identify those with ADHD early and accurately. Second, it is important to train teachers in how to apply the scale. Third, the scale should be used to identify the quality of educational interventions provided to students with ADHD. Finally, there is a need for a study to identify the prevalence ADHD, as described by the *DSM-5*, among children and adolescents in Sudan.

## Data Availability Statement

The original contributions presented in the study are included in the article/Supplementary Material, further inquiries can be directed to the corresponding author.

## Ethics Statement

The studies involving human participants were reviewed and approved by King Saud University. The patients/participants provided their written informed consent to participate in this study.

## Author Contributions

AA, AAA, DB, RA, SB, MJ, MA, NE, and NA contributed to the design and implementation of the study, to the collection and analysis of the data, to the presentation and discussion of the results, and the writing and finalizing the manuscript. All authors contributed to the article and approved the submitted version.

## Conflict of Interest

The authors declare that the research was conducted in the absence of any commercial or financial relationships that could be construed as a potential conflict of interest.

## Publisher’s Note

All claims expressed in this article are solely those of the authors and do not necessarily represent those of their affiliated organizations, or those of the publisher, the editors and the reviewers. Any product that may be evaluated in this article, or claim that may be made by its manufacturer, is not guaranteed or endorsed by the publisher.

## Footnotes

^1^Fathy, J. (2010). *Behavioral Disorders (Aggressive Behavior, Hyperactivity, Rebellion from school) among basic stage Pupils: A Comparative Study of public, Private, and model Schools in Khartoum Locality [Unpublished master’s thesis]*. Omdurman Islamic University, Sudan.

^2^Al Hassan, S. (2017). *The Effectiveness of a program to Enhance Self-Confidence for children with Learning Disabilities with Attention Deficit Hyperactivity Disorder in Khartoum Locality [Unpublished master’s thesis]*. University of Khartoum, Sudan.

^3^Al-Hussein, M. (2015). *Attention Deficit Hyperactivity Disorder and its Relationship to parental Treatment styles: A Descriptive Study on Parents of first-cycle children in Khartoum East Locality [Unpublished master’s thesis]*. Sudan: University of Science and Technology.

^4^Abdel-Qader, K. (2018). *The Effectiveness of a Behavioral program to reduce Attention Disorder Accompanied by Hyperactivity for Students with Learning Difficulties in Integrated Excellence Schools in Bahri Locality [Unpublished master’s thesis]*. Khartoum state: Sudan University of Science and Technology.
