# Supplementary material for: Psychometric Properties of ADHD Rating Scale—5 for Children and Adolescents in Sudan—School Version
Source: Front Psychol. 2022 Jun 27;13:883578. doi: 10.3389/fpsyg.2022.883578 (PMC9272821; doi:10.3389/fpsyg.2022.883578)
Supplement: Supplementary file 1 [file Table_1.doc]

# Appendix

**Table A1**

*Descriptive statistic for symptom items of the ADHD Rating Scale-5*

| **Item** | ***N*** | ***M*** | ***S.E.*** | ***LL*** | ***UL*** | ***SD*** |
| --- | --- | --- | --- | --- | --- | --- |
| Attention to details | 3742 | 0.44 | 0.012 | 0 | 3 | 0.734 |
| Sustaining attention | 3742 | 0.46 | 0.012 | 0 | 3 | 0.740 |
| Does not seem to listen | 3742 | 0.44 | 0.012 | 0 | 3 | 0.718 |
| Follow instructions | 3741 | 0.49 | 0.013 | 0 | 3 | 0.785 |
| Difficulty organizing | 3742 | 0.50 | 0.013 | 0 | 3 | 0.787 |
| Sustained mental effort | 3740 | 0.52 | 0.013 | 0 | 3 | 0.799 |
| Loses things | 3742 | 0.52 | 0.013 | 0 | 3 | 0.784 |
| Distracted | 3742 | 0.59 | 0.014 | 0 | 3 | 0.830 |
| Forgetful | 3742 | 0.49 | 0.013 | 0 | 3 | 0.786 |
| Inatt. total | 3742 | 4.45 | 0.089 | 0 | 27 | 5.426 |
| Fidgets | 3742 | 0.36 | 0.011 | 0 | 3 | 0.686 |
| Leaves seat | 3742 | 0.42 | 0.012 | 0 | 3 | 0.739 |
| Runs about | 3742 | 0.38 | 0.012 | 0 | 3 | 0.706 |
| Playing quietly | 3742 | 0.38 | 0.011 | 0 | 3 | 0.700 |
| On the go | 3742 | 0.44 | 0.013 | 0 | 3 | 0.767 |
| Talks excessively | 3741 | 0.53 | 0.013 | 0 | 3 | 0.814 |
| Blurts out answers | 3740 | 0.48 | 0.013 | 0 | 3 | 0.770 |
| Awaiting turns | 3742 | 0.44 | 0.012 | 0 | 3 | 0.743 |
| Interrupts or intrudes | 3742 | 0.37 | 0.011 | 0 | 3 | 0.699 |
| Hyp.-Imp. total | 3742 | 3.80 | 0.081 | 0 | 27 | 4.957 |
| Teach. ratings tot. | 3742 | 8.25 | 0.153 | 0 | 54 | 9.365 |

*Note*. *N* varies due to missing/false values.

**Table A2**

*Descriptive statistic for impairment items of the ADHD Rating Scale-5*

| **Item** | ***N*** | ***M*** | ***S.E.*** | ***LL*** | ***UL*** | ***SD*** |
| --- | --- | --- | --- | --- | --- | --- |
| Teacher relations | 3742 | 0.36 | 0.012 | 0 | 3 | 0.705 |
| Peer relations | 3742 | 0.34 | 0.011 | 0 | 3 | 0.678 |
| Academic funct. | 3742 | 0.52 | 0.013 | 0 | 3 | 0.821 |
| Behavioral funct. | 3742 | 0.51 | 0.013 | 0 | 3 | 0.814 |
| Homework funct. | 3742 | 0.35 | 0.011 | 0 | 3 | 0.701 |
| Self-Esteem | 3742 | 0.32 | 0.011 | 0 | 3 | 0.664 |
| After Inatt. tot. | 3742 | 2.39 | 0.056 | 0 | 18 | 3.402 |
| Teacher relations | 3742 | 0.29 | 0.010 | 0 | 3 | 0.638 |
| Peer relations | 3742 | 0.30 | 0.010 | 0 | 3 | 0.623 |
| Academic funct. | 3742 | 0.45 | 0.013 | 0 | 3 | 0.782 |
| Behavioral funct. | 3742 | 0.43 | 0.012 | 0 | 3 | 0.762 |
| Homework funct. | 3742 | 0.32 | 0.011 | 0 | 3 | 0.683 |
| Self-Esteem | 3742 | 0.27 | 0.010 | 0 | 3 | 0.623 |
| After Hyp.-Imp. tot. | 3742 | 2.07 | 0.053 | 0 | 18 | 3.218 |
| Impairment total | 3742 | 4.45 | 0.033 | 0 | 36 | 2.000 |
